# Systemic Preconditioning by a Prolyl Hydroxylase Inhibitor Promotes Prevention of Skin Flap Necrosis via HIF-1-Induced Bone Marrow-Derived Cells

**DOI:** 10.1371/journal.pone.0042964

**Published:** 2012-08-07

**Authors:** Mitsuru Takaku, Shuhei Tomita, Hirotsugu Kurobe, Yoshitaka Kihira, Atsushi Morimoto, Mayuko Higashida, Yasumasa Ikeda, Akira Ushiyama, Ichiro Hashimoto, Hideki Nakanishi, Toshiaki Tamaki

**Affiliations:** 1 Department of Plastic and Reconstructive Surgery, The University of Tokushima Graduate School, Tokushima, Japan; 2 Department of Pharmacology, The University of Tokushima Graduate School, Tokushima, Japan; 3 Department of Cardiovascular Surgery, The University of Tokushima Graduate School, Tokushima, Japan; 4 Nutrition and Metabolism, Institute of Health Biosciences, The University of Tokushima Graduate School, Tokushima, Japan; 5 Department of Environmental Health, National Institute of Public Health, Saitama, Japan; University of Padova, Italy

## Abstract

**Background:**

Local skin flaps often present with flap necrosis caused by critical disruption of the blood supply. Although animal studies demonstrate enhanced angiogenesis in ischemic tissue, no strategy for clinical application of this phenomenon has yet been defined. Hypoxia-inducible factor 1 (HIF-1) plays a pivotal role in ischemic vascular responses, and its expression is induced by the prolyl hydroxylase inhibitor dimethyloxalylglycine (DMOG). We assessed whether preoperative stabilization of HIF-1 by systemic introduction of DMOG improves skin flap survival.

**Methods and Results:**

Mice with ischemic skin flaps on the dorsum were treated intraperitoneally with DMOG 48 hr prior to surgery. The surviving area with neovascularization of the ischemic flaps was significantly greater in the DMOG-treated mice. Significantly fewer apoptotic cells were present in the ischemic flaps of DMOG-treated mice. Interestingly, marked increases in circulating endothelial progenitor cells (EPCs) and bone marrow proliferative progenitor cells were observed within 48 hr after DMOG treatment. Furthermore, heterozygous HIF-1α-deficient mice exhibited smaller surviving flap areas, fewer circulating EPCs, and larger numbers of apoptotic cells than did wild-type mice, while DMOG pretreatment of the mutant mice completely restored these parameters. Finally, reconstitution of wild-type mice with the heterozygous deficient bone marrow cells significantly decreased skin flap survival.

**Conclusion:**

We demonstrated that transient activation of the HIF signaling pathway by a single systemic DMOG treatment upregulates not only anti-apoptotic pathways but also enhances neovascularization with concomitant increase in the numbers of bone marrow-derived progenitor cells.

## Introduction

Local skin flaps are a general method of skin reconstruction in plastic surgery. However, flap necrosis caused by disruption of the blood supply within the flaps often occurs after the surgery, prolonging the treatment period and occasionally requiring a second operation. Therefore, alternative chemical treatments with secure flap elongation capacity would have clinical significance. To date, while a great deal of research on chemical delay has been reported, the effects of pharmacological augmentation of skin flap viability have been inconclusive.

Hypoxia-inducible factors (HIFs) are the key transcriptional factors in cellular adaptive responses to hypoxia. HIFs directly regulate the major angiogenic cytokines such as vascular endothelial growth factor (VEGF)-A, endothelial nitric oxide synthase (eNOS), angiopoietin (ANGPT), and platelet-derived growth factor (PDGF), and stromal cell-derived factor 1 (SDF-1) [1]. Moreover, HIFs regulate the metabolic switch to anaerobic glycolysis and apoptotic mechanisms [2]. HIFs thus play a pivotal prosurvival role in ischemic tissues. Therefore, there have been numerous reports of attempts to overcome ischemic conditions by stabilizing HIFs.

Local upregulation of HIF-1 promotes mobilization and recruitment of endothelial progenitor cells (EPCs) via direct upregulation of SDF-1 [3]. In contrast, stabilization of HIF-1 in EPCs contributes to direct or indirect angiogenesis and vasculogenesis through enhanced retention of EPCs in ischemic tissue and expression of growth factors such as PDGF [4], [5]. However, the effects of HIF stabilization on progenitor cells in the bone marrow remain unclear. Here we use the term “bone marrow-derived progenitor cells” (BMDPCs) to denote a heterogeneous population that includes EPCs, which incorporate into the endothelium of new or remodeling vessels, as well as myeloid, mesenchymal, and hematopoietic stem cells, which promote vascular growth and remodeling through production of angiogenic cytokines [6], [7], [8], [9], [10], [11], [12].

The hydroxylase activity of prolyl hydroxylase domain (PHD) proteins depends on the availability of O_2_, Fe(II), and 2-oxoglutarate. The PHD inhibitor dimethyloxalylglycine (DMOG), a 2-oxoglutarate analogue, inhibits the interaction between 2-oxoglutarate and PHDs, resulting in decreased PHD hydroxylase activity and stabilization of HIFs. PHD inhibitors have been used in some disease models, including limb ischemia [13] and diabetic ulcers [14]. Because the angiogenic and cytoprotective effects of PHD inhibitors are mediated by the HIF signaling pathway, combined inhibition of PHDs and stabilization of HIFs is an attractive therapeutic target. However, the clinical trials conducted thus far have shown conflicting results, and a method for clinical application of this strategy remains to be defined.

To test our hypothesis that stabilization of HIF-α proteins before skin flap surgery augments skin flap viability by promoting angiogenesis and tissue resistance to ischemia, we examined the effects of DMOG pretreatment in a mouse ischemic random pattern skin flap model. Our results indicate a possible new therapeutic strategy for treatment of patients with peripheral circulation insufficiency.

## Results

### DMOG pretreatment increases the survival area of ischemic skin flaps in mice

**Figure 1:**
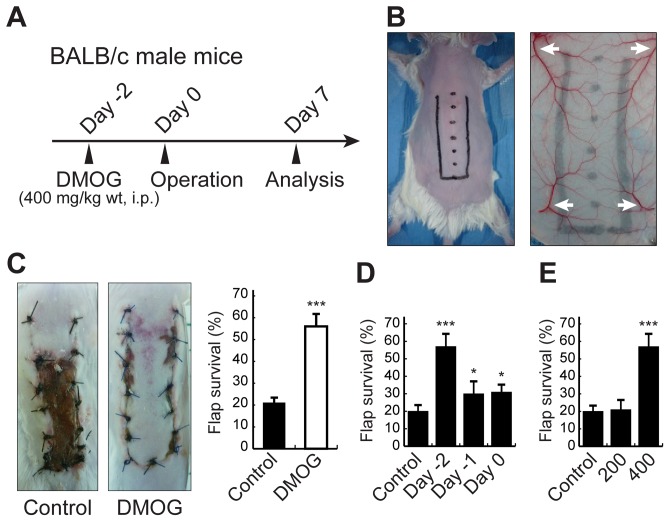
Effect of DMOG pretreatment on expanding survival area of ischemic skin flaps in mice. A, BALB/c mice were randomly assigned to the control group or the experimental group with intraperitoneal DMOG pretreatment (400 mg/kg body weight), followed by evaluation of flap survival on postoperative day 7. B, Design of ischemic random pattern skin flap and vascular distribution on the mouse dorsum. The flap was designed not to include any major pedicles arising from the deep circumflex iliac vessels and lateral thoracic vessels (white arrows). The distal parts of the flaps exhibited tissue necrosis due to disruption of the blood supply. C, Percentages of the survival area of the flap and dividing by the total area of the flap. Representative ischemic flaps show grossly better flap survival in the DMOG group. D, Effects of timing of DMOG pretreatment on the expansion of flap survival. DMOG pretreatment was performed 2 days or 1 day before the surgery or on the day of the surgery. E, Effect of dose of DMOG pretreatment on the expansion of flap survival. DMOG pretreatment was performed 2 days prior to surgery. The percentages of flap survival ± SEM were measured on postoperative day 7. *P>0.05; ***P>0.001.

To assess the effects of DMOG pretreatment on skin flap survival, we used a dorsal random pattern skin flap model (Figure 1A and B, Details described in Materials and Methods section). This model produced severe necrosis of the distal parts of the skin flaps. At postoperative day 7, the flap survival area was significantly larger in the DMOG-treated mice, especially at the distal part of the flap, than in the controls. Quantitative analyses showed that the flap survival area was 55.5±13.2% of the total area in the DMOG-treated group but only 20.6±4.9% in the untreated group (*P*<0.001; Figure 1C).

To test the conditions under which DMOG pretreatment was effective for promoting flap survival, we examined the time- and dose- dependences of DMOG administration in the operated mice. Mice pretreated with DMOG 2 days before surgery exhibited greater expansion of the flap survival area than those treated 1 day before or on the day of the surgery (Figure 1D, 56.8±14.3% compared with 32.1±5.1% and 30.6±8.0%, respectively). A dose of 400 but not 200 mg/kg body weight DMOG was sufficient to increase the flap survival area in treated mice relative to the control group (Figure 1E, 56.8±14.3% and 21.7±6.6% in the DMOG-treated groups, respectively). Therefore, the subsequent experiments in this study were performed using pretreatment with DMOG at 400 mg/kg body weight 2 days before surgery.

### DMOG pretreatment enhances postoperative angiogenesis

**Figure 2:**
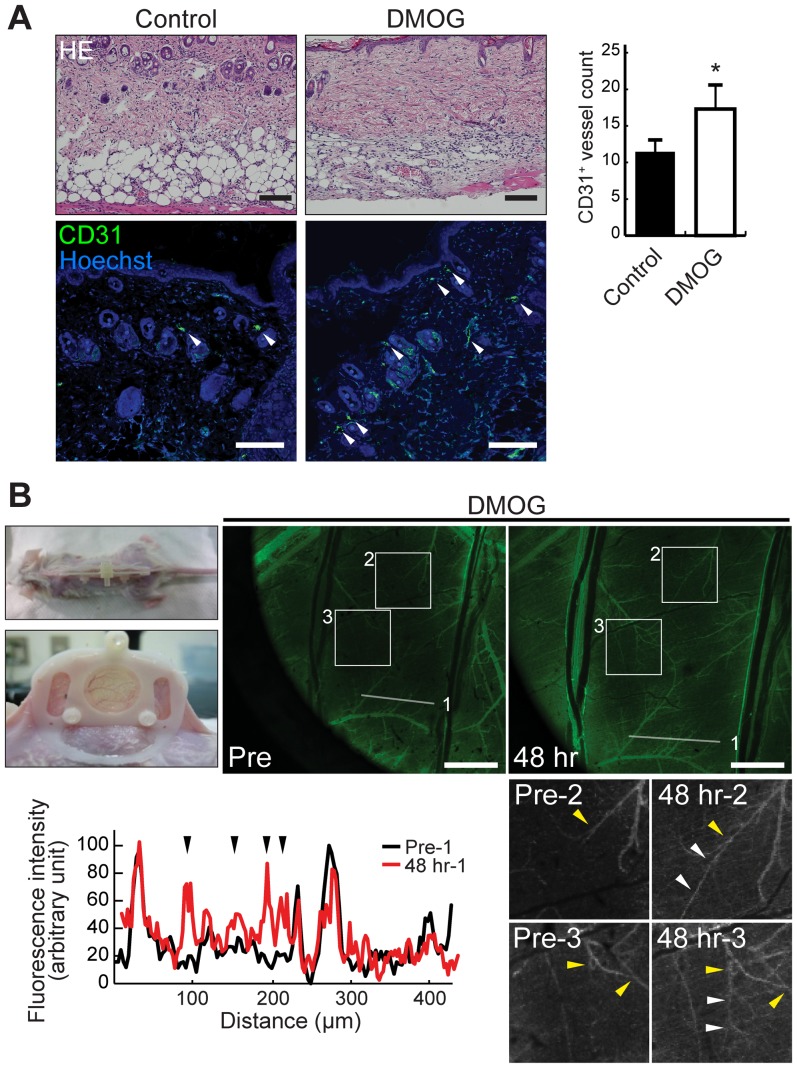
Effects of DMOG treatment on postoperative angiogenesis and vasculature in mouse subdermal plexus. A, Sections of proximal parts of the skin flaps near the pedicles were stained with hematoxylin and eosin (HE) and with anti-CD31 antibody (arrowheads). To evaluate the effect of DMOG on the neovascularization in the proximal part of the flap, the number of CD31-positive (green) vessels was counted and is indicated as vessel density per high-power field. Scale bar indicates 200 µm. B, Intravital microscopic analysis of the vasculature of the subdermal plexus in DMOG-treated mice. After the intraperitoneal injection of DMOG, the subdermal plexus visualized by intravenous injection of FITC-dextran was observed in the dorsal skin-fold chamber by fluorescence microscope. Fluorescent intensity at cross-section of the line 1 showed that the number of detectable vessels was increased 48 hours after DMOG treatment, compared to the untreated control (Indicators of arrowheads in the graph represent the detectable vessels). Magnified images of square frame line 2 and 3 showed that vasodilation of preexisting vessels observed in the border zone of the vascular territory. Yellow arrowheads indicate the relative locations in the vasculature as the landmarks, and white arrowheads the detectable vessels in the border zone of the vascular territory 48 hours after DMOG treatment. Scale bar indicates 250 µm.

Because the major source of flap necrosis is generally the failure of the blood supply, we evaluated the number of vessels in the proximal part of the skin flap on postoperative day 7. The number of CD31-positive vessels was significantly greater in the DMOG group than in the control group (17.3±3.3 vs. 11.3±1.7%, P<0.05; Figure 2A). Because the blood and nutrition within random skin flaps are supplied by marginal vessels at the edges of the vascular territory, we used dorsal skin fold chambers to sequentially observe the preexisting vessels in the subdermal plexus in DMOG-treated mice. Significant enhancement in vasodilation of preexisting vessels was observed in the border zone of the vascular territory 48 hours after DMOG treatment (Figure 2B).

### DMOG pretreatment induces HIF-1α protein expression in the skin both pre- and postoperatively

**Figure 3:**
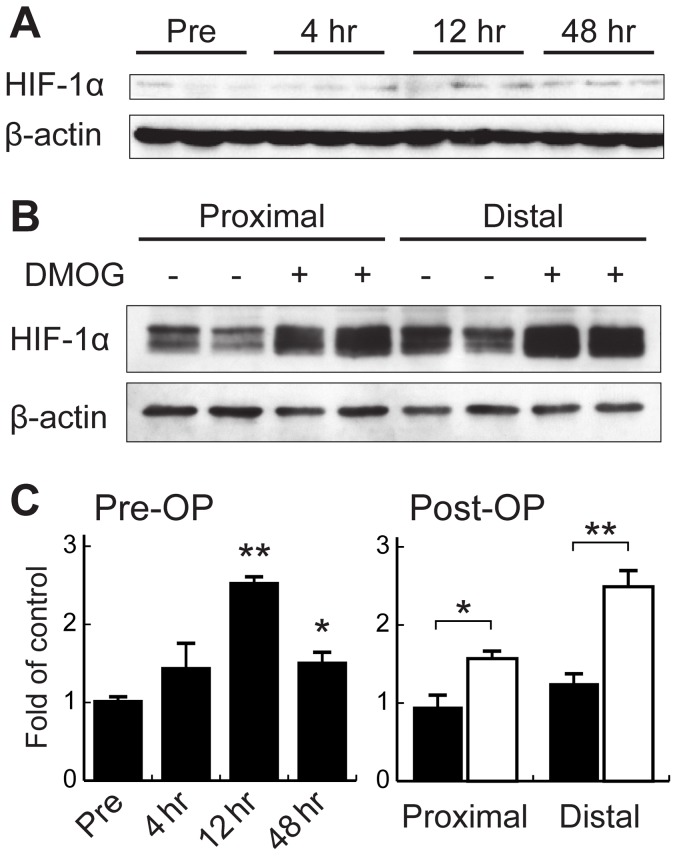
Expression of HIF-1α protein in the skin after intraperitoneal administration of DMOG. Immunoblot analyses were performed on tissue lysates from dorsal skin taken preoperatively (A and C) and from the proximal and distal parts of the skin flaps on postoperative day 1 (B and C). In panel A and C, HIF-1α protein expression in the skin is shown before (Pre) and at 4, 12, and 48 hours after DMOG treatment. *P<0.05 and **P<0.01 compared with Pre. In panel B and C, *P<0.05 and **P<0.01 compared with the untreated mice. β-Actin was used as a loading control.

To test the effect of systemic administration of DMOG on stabilization of HIF-1α protein in the skin, we excised the dorsal skin at different time points pre- and postoperatively and performed Western blot analysis. The level of HIF-1α protein expression was maximally increased, to 2.5-fold over the pretreatment level, 12 hours after DMOG treatment. The expression was still upregulated at the time of the flap operation 48 hrs after DMOG administration (Figure 3A and C). Furthermore, 1 day after the skin flap operation, the expression of HIF-1α protein was markedly increased especially in distal part of the flap region, and was detected as two bands of HIF-1α proteins partially modified by phosphorylation during hypoxic conditions [15] (Figure 3B and C, 1.5- and 1.9-fold over the control in the proximal and distal parts, respectively). These data suggest that systemic DMOG pretreatment allows HIF-1α protein to accumulate in mouse skin and that this protein accumulation continues in the skin flap region even a day after the operation.

### DMOG pretreatment induces angiogenic cell mobilization early after the flap operation

**Figure 4:**
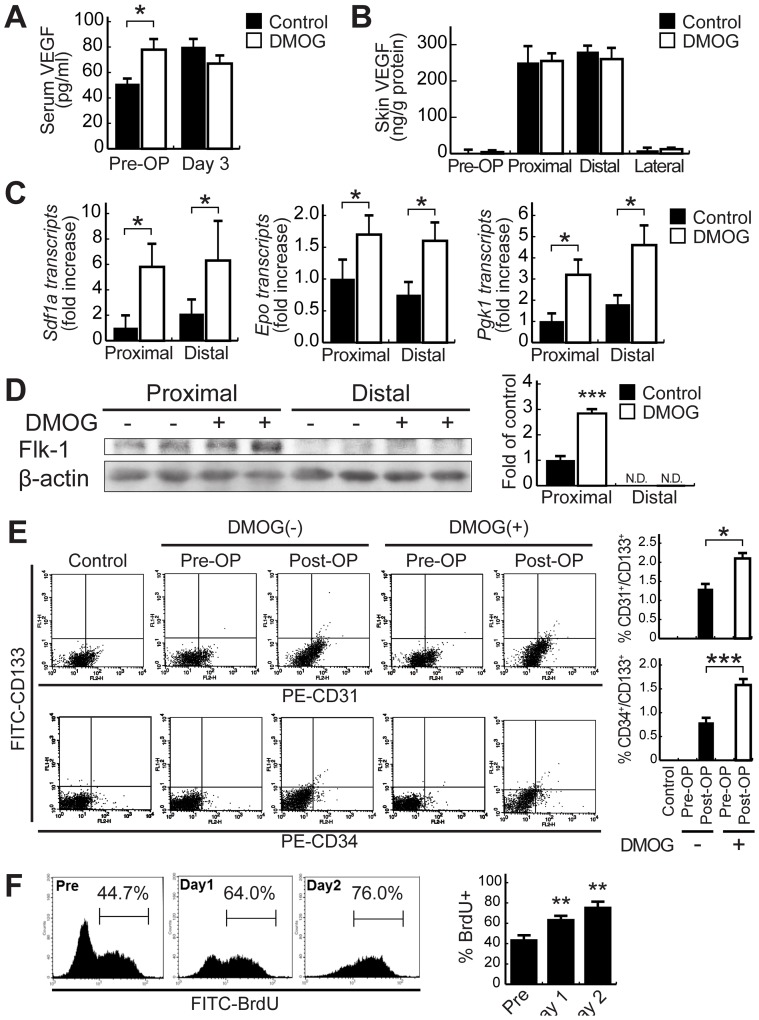
Effects of DMOG pretreatment on angiogenesis-related factors in the mouse skin flap model. A, Serum VEGF concentrations before and after surgery on DMOG-treated or untreated mice were measured by ELISA. Pre-OP and Day 3 indicate pre-surgery and on day 3 after the surgery, respectively. B, VEGF proteins in the skin tissues before and after surgery were measured by ELISA. Skin tissues from the proximal, distal, and lateral parts of the skin flaps were prepared for the assay. C and D, Transcript levels of HIF-1 target genes (C) and the expression of FLK-1 proteins (D) in the proximal and distal parts of the skin flaps on postoperative day 1 were assessed by quantitative RT-PCR and Western blot analysis, respectively. N.D. indicates not detectable. Values are means ± SDM. *P<0.05.E, Left panel, the representative flow cytometric profiles of peripheral blood cells from DMOG-treated or untreated mice. Peripheral blood cells were stained with anti-CD133, anti-CD45, anti-CD34, and anti-CD31 antibodies and analyzed with a flow cytometer. Pre-OP and Post-OP indicate before the flap operation and 1 day after the flap operation, respectively. Data were representative of at least 4 independent experiments. Right panel, summary of the ratio of the EPCs (gated on CD45-positive cells) in peripheral blood cells. F, Cell proliferation in bone marrow cells (gated on CD45-positive and CD34-positive cells) from untreated or DMOG-treated mice 1 and 2 days after the treatment was monitored by BrdU incorporation and analyzed by flow cytometry. *P<0.05, ***P<0.001.

To investigate whether DMOG could induce mobilization of angiogenic cells from the bone marrow, we next examined the effects of DMOG pretreatment on angiogenesis-related factors in the mouse skin flap model. The circulating VEGF concentration was greater in the DMOG-treated mice than in the untreated mice on the day before surgery (52.34±1.64 vs. 77.9±4.49 pg/ml, *P*<0.05; Figure 4A), but the treated and untreated mice exhibited similar levels on postoperative day 3. In addition, VEGF concentrations in the proximal and distal skin flap tissues increased dramatically after the surgery, but similar increases were observed in the DMOG-treated and untreated mice (Figure 4B). In contrast, transcript levels of some other HIF-1 target molecules such as SDF-1, a chemokine for angiogenesis by recruiting EPCs from the bone marrow, erythropoietin (EPO) and phosphoglycerate kinase 1 (PGK1), a glycolytic enzyme, were significantly increased in both the proximal and distal skin flap tissues (Figure 4C). In addition, immunoblot analysis showed a significant increase in FLK-1 (also named VEGFR-2) expression in the proximal part of the flap on postoperative day 1; this effect was enhanced in the DMOG-treated group (Figure 4D). The FLK-1 expression level in the distal part of the flap was hardly detectable by immunoblot assay despite the fact that the VEGF concentration was elevated in the distal part. These results indicate that the recovery of blood circulation occurs sequentially from the pedicle of the flap to the severely ischemic distal part of the flap and depends on local skin flap characteristics.

Because recruitment of bone marrow-derived cells, including endothelial progenitor cells (EPCs), has been demonstrated to be involved in the neovascularization of ischemic tissues [16], we next evaluated the presence of EPCs as a marker of vasculogenesis by flow cytometry using the cell surface markers CD45, CD133, CD34, and CD31. Here EPCs were defined as CD133^+^CD45^+^CD34^+^ or CD133^+^CD45^+^CD31^+^ cells to detect early responding and specific EPCs to the skin flap operation. We detected these EPCs gated with CD45-positive cells to eliminate late EPC population and CD31-positive cells to contain more specific EPC population [17], [18]. The percentages of EPCs in the peripheral blood were significantly greater in the DMOG-treated group than in the control group (1.28±0.17 vs. 2.2±0.2% CD45^+^CD133^+^CD31^+^ cells, *P*<0.005; 0.74±0.03 vs. 1.46±0.13% CD45^+^CD133^+^CD34^+^ cells, *P*<0.05; Figure 4E) on post-operative day 1, whereas these cells were barely detectable immediately prior to the surgery, 48 hr after the single DMOG treatment. We hypothesized that systemic administration of DMOG may increase proliferation of angiogenic cells in the bone marrow, resulting in elevated levels of circulating EPCs in the early postoperative phase. We therefore investigated cell proliferation in the bone marrow of DMOG-treated and untreated mice using 5-bromo-2′-bromodeoxyuridine (BrdU)-incorporation assays and flow cytometry. Bone marrow cells were isolated 1 hour after intraperitoneal injection of BrdU. The percentage of BrdU-positive cells in the CD45^+^CD34^+^ hematopoietic progenitor cell population, which should include the EPCs, was measured. As expected, the percentages of dividing CD45^+^CD34^+^ cells (i.e., those incorporating BrdU) in the bone marrow 2 days after DMOG treatment were significantly greater, constituting up to 75% of the bone marrow cells, in the DMOG-treated group than in the untreated group (1.55-fold over control, *P*<0.05; Figure 4F).

### DMOG pretreatment suppresses apoptosis in the distal part of the skin flap

**Figure 5:**
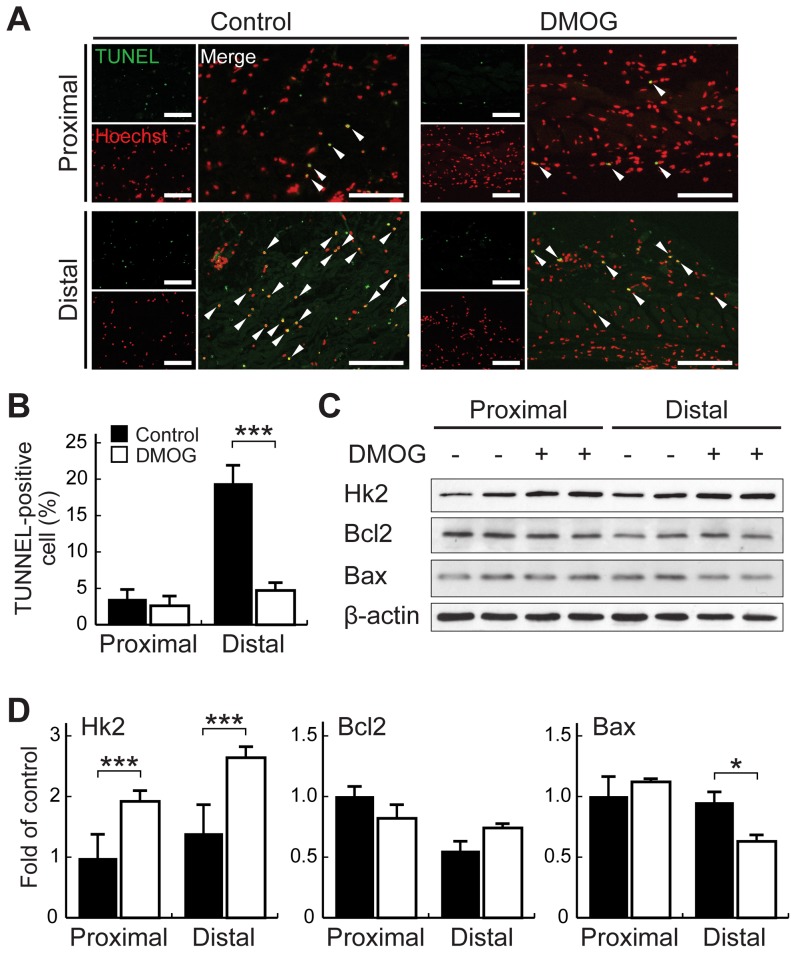
Effects of DMOG pretreatment on apoptosis in the mouse skin flap model. A, The apoptotic cells in the proximal and distal parts of the skin flaps taken on postoperative day 1 from mice with or without DMOG pretreatment were detected by TUNEL staining. Sections of the skin flaps were stained for TUNEL (green) and Hoechst (red). Scale bar indicates 100 µm. B, The ratio of TUNEL-positive cells to cell nuclei in the sections was calculated. Values are means ± SEM. ***P<0.001. C and D, Evaluation of expression of apoptosis-related factors in BALB/c mice. HK2, BCL2, and BAX protein expression levels in the proximal and distal parts of the skin flaps harvested on postoperative day 1 were detected by immunoblotting of protein extracts.

Because the distal parts of flaps must resist severe ischemia until the blood supply to this ischemic region recovers, we used the ischemic skin flap model to assess whether DMOG pretreatment suppresses apoptosis via the accumulation of HIF-1α protein. Apoptotic cells were detected by TUNEL staining of the proximal and distal parts of the skin flaps of mice with or without DMOG pretreatment examined on postoperative day 1. In the proximal parts of the ischemic flap tissue sections, the percentages of TUNEL-positive cells were similar between the DMOG-treated and untreated groups (2.72±1.08% vs. 3.71±0.97%, *P*>0.05; Figure 5A and B). In contrast, the distal parts of the ischemic flaps in the DMOG-treated group contained significantly fewer apoptotic cells than did those of the untreated group (4.76±0.87% vs. 19.47±2.15%, *P*<0.001; Figure 5A and B). As shown in Figure 5C, the expression levels of pro- and anti-apoptotic factors were assessed by Western blot analysis on postoperative day 1. The levels of anti-apoptotic BCL-2 proteins in both the proximal and distal parts of the skin flaps were not increased by DMOG pretreatment compared with the untreated controls. However, DMOG pretreatment significantly reduced expression of the pro-apoptotic BAX proteins, indicating that the observed reduction of apoptotic cells in the distal parts of the flaps was due to the shift in the Bcl-2/Bax ratio. In addition, DMOG treatment increased the expression of hexokinase 2 (Hk2), a primary initiator of glycolysis that binds mitochondria to inhibit Bax-induced cytochrome c release and apoptosis [19], in both the proximal and distal parts of the skin flaps relative to those of the untreated group (Figure 5D). These results suggest that DMOG treatment suppresses ischemia-induced apoptosis in the distal part of the skin flap.

### Survival of ischemic skin flaps is impaired by Hif-1α haploinsufficiency

**Figure 6:**
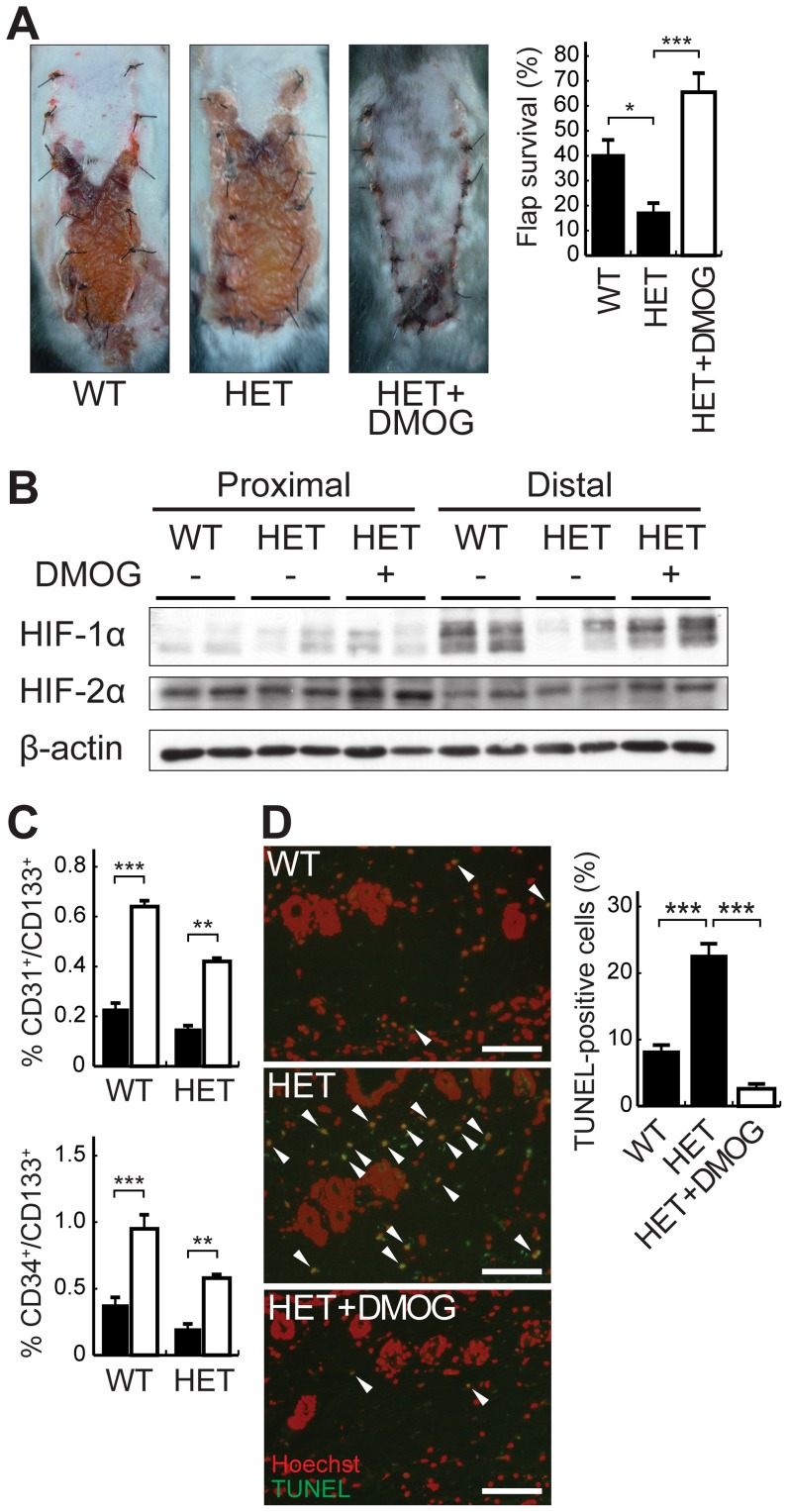
Effects of loss of HIF-1α function on flap survival. A, Representative ischemic skin flaps from wild-type (WT), heterozygous HIF-1α deficient (HET), and HET with DMOG pretreatment on postoperative day 7. B, Evaluation of the expression of HIF-1α and HIF-2α proteins in the proximal and distal parts of ischemic skin flaps on postoperative day 1. C, Measurement of EPCs in peripheral blood on postoperative day 1. DMOG pretreatment increased the numbers of EPCs in both WT and HET mice. The percentage of the peripheral blood cells constituted by the EPC population was measured as the percentage of CD45^+^-gated cells positive for the indicated cell surface markers by flow cytometry. D, The apoptotic cells in the distal parts of the skin flaps taken from WT and HET mice with or without DMOG pretreatment on postoperative day 1 were detected by TUNEL staining. Sections of the skin flaps were stained for TUNEL (green) and Hoechst (red). The ratio of TUNEL-positive cells to cell nuclei in the sections was calculated. Values are means ± SEM. *P<0.05, **P<0.01, ***P<0.001. Scale bar indicates 50 µm.

To confirm that the effects of DMOG on flap survival were via an HIF-1α signaling pathway, we examined the survival of ischemic skin flaps in heterozygous HIF-1α-deficient (HET) mice [20]. Compared with their wild type (WT) littermates, HET mice showed significantly reduced areas of flap survival (40.05±4.89 vs. 17.05±2.98%, *P*<0.05; Figure 6A). This reduction of flap survival in HET mice was significantly restored by DMOG pretreatment (17.05±2.98% for untreated mice vs. 64.21±6.92% for DMOG-treated mice, P<0.001; Figure 6A). We confirmed the effects of DMOG on the expression of HIFs in HET mice and found that the expression of HIF-1α of ischemic flaps was lower in HET mice than in WT mice and was restored, especially in the distal parts of the flaps, by DMOG pretreatment (Figure 6B). The levels of HIF-2α protein in the proximal and distal parts of the ischemic flaps of HET mice were similar to those of WT mice and were increased by DMOG pretreatment (Figure 6B). Interestingly, the tendency was for HIF-2α protein to be more highly expressed in the proximal parts of the flaps than in the distal parts, although the expression of HIF-1α protein was higher in the distal parts, which are more severely ischemia (Figure 6B). We next examined the percentages of EPCs in peripheral blood from WT and HET mice with or without DMOG treatment. DMOG pretreatment significantly increased the percentages of EPCs in the peripheral blood cells from both WT and HET mice on postoperative day 1 (Figure 6C). The percentages of EPCs seemed to be higher in DMOG-treated HET mice than in untreated WT mice. In addition, while the percentages of EPCs were not significantly different between WT and HET mice, the HET mice appeared to have lower levels of EPCs (Figure 6C). TUNEL staining for detection of apoptotic cells in the distal parts of the flaps demonstrated that the numbers of apoptotic cells on postoperative day 1 were significantly greater in HET mice than in WT mice (8.08±0.79% vs. 22.37±1.43%, P<0.001; Figure 6D), and DMOG pretreatment of HET mice inhibited apoptosis (2.5±0.5% vs. 22.37±1.43%, P<0.001; Figure 6D).

### HIF-1α expression in both the ischemic skin tissue and bone marrow cells has protective effects on skin flap necrosis

**Figure 7:**
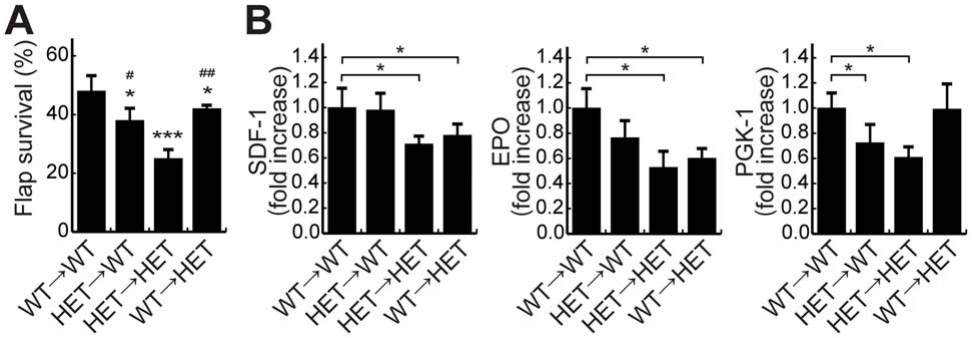
Effect of bone marrow cell expression of HIF-1α on skin flap survival. A, Percentages of skin flap survival were calculated in WT and HET mice reconstituted with bone marrow from either WT or HET donor mice. Values are means ± SEM. *P<0.05, ***P<0.001 vs. WT reconstituted with bone marrow from WT mice. ^#^ *P*<0.05, ^##^ *P*<0.01 vs. HET reconstituted with bone marrow from HET mice. B, Transcript levels of HIF-1 target genes were assessed by qRT-PCR analysis at the ischemic skin flap on postoperative day 1 in bone marrow cell-transplanted mice (n = 6). Values are means ± SDM. *P<0.05.

Systemic administration of DMOG increased the number of EPCs in peripheral blood and the proliferation of bone marrow progenitor cells. To determine whether HIF-1α expression in bone marrow cells contributes to the survival of ischemic skin flaps, we grafted irradiated WT or HET mice with WT or HET bone marrow cells and performed flap surgery 4 weeks after bone marrow transplantation. Compared with WT mice reconstituted with WT bone marrow cells, WT mice reconstituted with HET bone marrow cells had significantly lower skin flap survival areas (48.27±4.30% vs. 38.15±6.77%, *P*<0.05; Figure 7A). Moreover, HET mice reconstituted with WT bone marrow cells had significantly greater flap survival than HET mice reconstituted with HET bone marrow cells (42.63±0.16% vs. 25.91±2.08%, *P*<0.01; Figure 7A). Compared with WT mice reconstituted with WT or HET bone marrow cells, HET mice reconstituted with WT or HET bone marrow cells had significantly lower flap survival (48.27±4.30% vs. 42.63±0.16%, *P*<0.05 and 38.15±3.38% vs. 25.91±2.08%, *P*<0.05; Figure 7A). Transcript levels of some HIF-1 target genes such as Sdf1a, Epo and Pgk1 were examined in the ischemic skin flap tissues, and showed that the expressions of SDF-1 and EPO were significantly decreased in HET mice reconstituted with the bone marrow cells, regardless of genotype in the donor mice (Figure 7B). On the other hand, the expression of PGK1 was significantly decreased in mice reconstituted with HET bone marrow cells, regardless of genotype in the host mice (Figure 7B). These data indicated that HIF-1α expression not only in the ischemic area but also in bone marrow cells significantly influences flap survival.

Taken together, these data suggest that DMOG-induced HIF-1α activity contributes greatly to the survival of ischemic skin flaps by increasing the numbers of bone marrow-derived angiogenic cells in the peripheral blood and suppressing apoptosis in the distal parts of the flaps.

## Discussion

Necrotic area caused by the skin flap operation should be determined at least in part by the relationship between the timing of neovasculogenesis and resistance to cell death in the ischemic tissues. In this study, we demonstrated that preconditioning of mice with DMOG, a PHD inhibitor, preserves skin flap tissue viability by both enhancing anti-apoptotic pathways and accelerating reconstitution of vessels in ischemic skin flaps. We showed for the first time that systemic administration of DMOG increases the number of progenitor cells in the bone marrow via the activation of HIF-1α, which is followed by reconstitution of the vasculature in the ischemic skin flap. Furthermore, we performed bone marrow transfer experiments using HIF-1-deficient mice and showed a possibility that the activation of HIF-1 signaling in these BMDPCs is a main event in the reconstruction of the vasculature in the ischemic tissue.

The HIF-mediated effects of PHD inhibitors on apoptosis in ischemia have been studied in acute renal failure [21] and a colitis model in vitro [22]. Moreover, stabilization of HIF-1α leads to enhanced protein-protein interactions between Hk2 and the voltage-dependent anion channel (VDAC), resulting in anti-apoptotic effects [23]. Our present data further indicate that stabilization of HIF-1α in response to DMOG treatment under severely ischemic conditions, i.e., in the distal parts of ischemic skin flaps, inhibits apoptosis through upregulation of Hk2, and the results from the HIF-1α-deficient mice support the critical protective role of HIF-1α and the sufficient protective effect of DMOG against severe ischemia.

We showed that systemic administration of DMOG by intraperitoneal injection 48 hours prior to the skin flap operation was sufficient for protection against ischemia. Maximal protection was observed with treatment 48 hours before but not 24 hours before or on the day of the operation. Therefore, some functional changes in the skin or the mice during the 48 hours after the administration of DMOG seemed to be prerequisite for this protective effect. The necessity of a 48-hour pretreatment period for the protective effect led us to examine whether DMOG-induced vascular formation could be attributed to functional enhancement of BMDPCs by the activated HIF-1.

Recruitment of bone marrow cells has been demonstrated to be involved in neovascularization of ischemic tissues [24], [25]. A higher revascularization potential of circulating angiogenic cells (CACs), including EPCs or HIF-activated CACs, has been reported in a mouse model of limb ischemia [4], [5], [26], [27], [28] and wound healing [29]. Our data also showed that systemic administration of DMOG enhanced the number of circulating EPCs observed after the initiation of the ischemic skin flap operation. Furthermore our data demonstrated, for the first time, that DMOG induces proliferation of progenitor cells in the bone marrow, presumably increasing the number of circulating EPCs, a subset of the BMDPC population, in the peripheral blood in the early postoperative phase. Therefore, BMDPCs are certain to be a critical site of action for the DMOG-induced protective effect on ischemic skin flap necrosis. Indeed, as shown in Figure 7, the bone marrow transfer experiments using heterogeneous HIF-1α-deficient mice showed that the expression of HIF-1α in bone marrow cells plays a significant role in the survival of ischemic skin flaps. Therefore, our results, in combination with the previous reports, suggest that systemic administration of DMOG acts through activation of HIF signaling pathways to enhance the number of progenitor cells in bone marrow, recruits them to the ischemic part of flap through the activation of chemotactic and growth signals such as SDF-1-CXCR4 [3] and VEGF-FLK-1 [25] pathways, and thereby reconstitutes the vasculature to reperfuse and regenerate the ischemic tissue.

We showed that DMOG treatment enhanced the number of circulating EPCs in our ischemic model. Recent reports have shown that HIF-1α-overexpressing EPCs were effective in treating ischemic tissues [4], [30]. However, throughout the past decade many other studies have shown only marginal importance for EPCs in models of ischemic or tumor-induced angiogenesis [31]. Bone marrow-derived cells, which have been demonstrated to be involved in promoting the reperfusion that is required to maintain tissue viability, include myeloid cells, mesenchymal cells, and hematopoietic stem/progenitor cells, as well as EPCs, [7], [10], [11], [16], [32]. Therefore, further study is required to identify the major subsets of the BMDPCs induced by DMOG treatment that contribute to the protection against ischemic necrosis. Identification of these BMDPC subsets will be beneficial in understanding the mechanism of DMOG-induced amelioration of ischemic tissue necrosis in order to design an effective therapeutic strategy for protection against such necrosis.

We also demonstrated that a single injection of DMOG in mice increases the number of CD34^+^CD45^+^ BMDPCs in the bone marrow within 48 hours and that these cells are critical for reconstitution of the vasculature in ischemic skin flaps. This effect of a single injection of DMOG is supported by a recent report that silencing of PHDs induced a transient upregulation of HIF-1α, leading to the promotion of revascularization in ischemic tissue [13]. In addition, while this work was in progress, another group reported that the presence of DMOG increased the number of CACs prepared from mouse bone marrow in an *in vitro* experiment [28]. This indicates that the enhancing effect of DMOG on revascularization in ischemic skin flaps may be mediated by activation of an intrinsic HIF signaling pathway in circulating BMDPCs. Taken together with other data, these findings indicate the potential clinical applications of the transient activation of HIF signaling pathways by a single systemic DMOG treatment as a new therapeutic strategy for ischemic preconditioning.

In conclusion, we reveal for the first time that transient activation of HIF signaling pathways in both ischemic skin tissue and bone marrow cells by a single systemic administration of a PHD inhibitor upregulates anti-apoptotic pathways and reconstitutes the vasculature in ischemic skin flaps, resulting in the preservation of ischemic tissue viability. This study also paves the way for future clinical strategies based on administration of PHD inhibitors to promote therapeutic tissue viability.

## Materials and Methods

### Ethics Statement

This study utilized experiments using mice. All experiments using mice were performed with consent from the Animal Experimentation Committee of the University of Tokushima (Toku Dobutu 11129; Committee members who approved this study are following, Hideyuki Nakagawa, Takuya Sasaki, Masayuki Azuma, Hiroyuki Fukui, Hideaki Nagamune, Mitsuru Matsumoto, Taku Okazaki, Hideki Nakanishi, Masato Mitome, Takahiro Matsumoto, Hiroshi Sakaue). The study did not involve human experiments.

### Animal Study

BALB/c mice (male, 10 weeks old, 25.53±0.86 g body weight), and heterozygous Hif-1α-deficient and wild-type littermate mice (male, C57BL/6J background (backcrossed more than ten times), 10–14 weeks old, 25.71±2.27 g body weight) were used in this study. Two experimental groups of each mouse strain (n = 6) were subjected to skin flap surgery. One group was administered DMOG (Cayman Chemical, Ann Arbor, MI) (400 mg/ml in saline) intraperitoneally 2 days before surgery (Figure 1A), while the other group received no treatment. A cranially based random pattern skin flap measuring 1.0×3.0 cm was elevated under the panniculus carnosus layer on the dorsum of each mouse (Figure 1B). The lateral thoracic vessels were ablated intraoperatively in order to establish the severely ischemic skin flap model. After flap elevation, the flap was sutured in place with 4-0 nylon and covered with Tegaderm (3M, Maplewood, MN). Seven days after surgery, the mice were anaesthetized and the area of skin flap survival measured.

### Skin Flap Survival Area Calculation

Pictures of the dorsal flaps were obtained using a digital camera (LUMIX DMC-FX150; Panasonic, Japan) on postoperative day 7. The necrotic area of the flap was determined grossly by the presence of scab formation, alopecia, and loss of elasticity. The survival area was calculated as a percentage of the total flap area using Image J Software.

### Western Blotting Analysis and ELISA

The ischemic flap measuring 1×3 cm was divided into 3 sections (proximal, middle, and distal parts). The proximal part measuring 1×1 cm was defined as a location adjacent to the pedicle of flap. Tissue samples from the proximal and distal parts were harvested on postoperative day 1. These samples were homogenized in lysis buffer containing 20 mmole/L Tris-HCl, pH 7.4, 150 mmole/L NaCl, 1 mmole/L EDTA, 1 mmole/L EGTA, 1% Triton X-100, 2.5 mmole/L sodium orthovanadate, 1 µg/ml leupeptin, and 1 mmole/L phenylmethyl sulfonyl fluoride. The samples were centrifuged to pellet the debris and the supernatants were analyzed. A volume of each extract corresponding to 25 µg of total protein was resolved on sodium dodecyl sulfate-polyacrylamide gels and electrotransferred to polyvinylidene difluoride membranes. The membranes were blocked in phosphate-buffered saline with 0.1% Tween-20 (PBS-T) containing 5% milk powder for 30 min at room temperature and then incubated overnight at 4°C with one of the following primary antibodies: anti-HIF-1α polyclonal antibody (Cayman Chemical, Ann Arbor, MI) at a dilution of 1∶400, anti-mouse FLK-1 polyclonal antibody (Santa Cruz Biotechnology, Santa Cruz, CA) at a dilution of 1∶200, anti-mouse HIF-2α antibody (R&D Systems, Minneapolis, MN) at a dilution of 1∶1000, anti-Bcl-2 (Cell Signaling Technology, Danvers, MA) at a dilution of 1∶1000, anti-Bax polyclonal antibody (Santa Cruz Biotechnology, Santa Cruz, CA) at a dilution of 1∶1000, anti-Hk2 monoclonal antibody (Cell Signaling Technology, Danvers, MA) at a dilution of 1∶1000, or anti-β-actin polyclonal antibody at a dilution of 1∶1000 as a loading control.

The membranes were subsequently incubated with horseradish peroxidase-conjugated anti-rabbit IgG (GE Healthcare Biosciences, Piscataway, NJ) or anti-goat IgG (Santa Cruz Biotechnology, Santa Cruz, CA) for 1 hr at room temperature, and the signals were developed using ECL Plus Western Blotting Detection System (GE Healthcare Biosciences, Piscataway, NJ).

Mouse blood was collected and allowed to clot for 30 min at 37°C and then centrifuged twice for 2 min at 5000×*g*. The serum was collected and stored at −80°C. Mouse skin was harvested on postoperative day 1. Tissue samples were homogenized in PBS and then stored at −80°C overnight. The homogenates were centrifuged for 5 min at 5000×*g* and the supernatants used for the ELISA assay. The ELISA assay was performed according to the manufacturer's protocol (R&D Systems, Minneapolis, MN).

### Histological Analysis and Immunohistochemistry

Tissues from the proximal and distal parts of the flaps collected on postoperative days 1 and 7 were fixed in 4% paraformaldehyde and embedded in paraffin. For histological evaluation or immunohistochemical staining for CD31, sections were deparaffinized in xylene and rehydrated in a series of ethanol washes. For detection of CD31-positive cells, sections were incubated with anti-mouse CD31 antibody (eBioscience, San Diego, CA) at a dilution of 1∶100 at 4°C overnight. For analysis of angiogenesis, the CD31-positive vessels in 10 fields were counted by light microscopy (×200) for each group. The In Situ Cell Death Detection Kit (Roche Diagnostics, Basel, Switzerland) was used to measure apoptosis of the flap by calculating the ratio of TUNEL-positive cells according to the manufacturer's protocol.

### Intravital Fluorescence Microscopy

The alterations of the subdermal plexus on the dorsum were observed by using dorsal skin-fold chamber (DSC) as previously described [33]. Ten-week-old male BALB/c mice were anesthetized (3 mg pentobarbital/100 g body weight), the dorsal skin was shaved and depilated, and the DSCs were implanted. The observation of the subdermal plexus was performed 5 days after DSC implantation. To visualize functional vessels, a total of 100 µl of fluorescein-5-isothiocyanate (FITC)-labeled dextran (MW 20,000,000, 50 mg/ml in 0.9% saline; Sigma, St. Louis, MO) was injected into the tail vein, and laser microscopy (AZ-C1; Nikon, Japan) at constant temperature was used for detection. The diameters of the functional vessels were observed sequentially in a continuous area, and Image J software (NIH, Besethsda, MD) was used to represent the diameters as pixels for comparison between the DMOG and control groups.

### Flow Cytometry Analysis of Cell Surface Markers

Peripheral blood was obtained from 10-week-old BALB/c mice and either heterozygous HIF-1α-deficient mice or wild-type littermates, with or without DMOG pretreatment, and the red blood cells were lysed with ammonium chloride. The remaining cells were labeled with either phycoerythrin (PE)-conjugated anti-CD31 (eBioscience, San Diego, CA) or biotin-conjugated anti-CD34 (eBioscience, San Diego, CA) with FITC-conjugated anti-CD133 (eBioscience, San Diego, CA) and allophycocyanin (APC)-conjugated anti-CD45 (eBioscience, San Diego, CA) and sorted by fluorescence-activated cell sorting (FACS).

Bromodeoxyuridine (BrdU) staining of bone marrow cells was performed using the BD Pharmingen™ BrdU Flow Kit according to the manufacturer's protocol (BD Biosciences Pharmmingen, San Diego, CA). BALB/c mice treated with or without DMOG received an initial intraperitoneal injection of BrdU (1 mg/mouse) 1 hour prior to sacrifice. Bone marrow cells were harvested from the femora and humeri. Samples were labeled with biotin-conjugated anti-CD34 (eBioscience, San Diego, CA), APC-conjugated anti-CD45 (eBioscience, San Diego, CA), and FITC-conjugated BrdU and sorted by flow cytometry.

### Mouse Bone Marrow Transplantation

Recipient HIF-1α^+/+^ (WT) and HIF-1α^+/−^ (HET) mice were lethally irradiated (9 Gy, γ-source) and reconstituted 24 hours later with bone marrow from either WT or HET donors. Skin flap surgery was performed 4 weeks after bone marrow transplantation.

### Statistical analysis

Values are expressed as means ± SEM for 3 separate experiments. Data were analyzed using a 1-way analysis of variance to determine significant differences among groups, after which a modified *t-*test with the Bonferroni correction was used for comparison between groups. *P* values<0.05 were accepted as statistically significant.
